# Preliminary Results of Sensorimotor Room Training for the Improvement of Sensory and Motor Skills in Children with Autism Spectrum Disorders

**DOI:** 10.3390/pediatric17010004

**Published:** 2025-01-08

**Authors:** Giulia Savarese, Rosa Mandia, Aldo Diavoletto, Michele Piscitelli, Francesca Impemba, Annatania Di Siervi, Luna Carpinelli, Franca Bottiglieri, Marianna Sessa, Giulio Corrivetti

**Affiliations:** 1 Department of Medicine and Surgery, University of Salerno, Baronissi Campus, 84081 Baronissi, Italy; lcarpinelli@unisa.it; 2 Cooperativa Giovamente, 84126 Salerno, Italy; rosamandia77@gmail.com (R.M.); michelepiscitelli88@gmail.com (M.P.); impembafra@gmail.com (F.I.); annataniadisiervi@gmail.com (A.D.S.); 3 ASL Salerno, 84124, Salerno, Italy; a.diavoletto@aslsalerno.it (A.D.); spec.bottiglieri@aslsalerno.it (F.B.); marianna.sessa10@gmail.com (M.S.); corrivetti@gmail.com (G.C.)

**Keywords:** autism spectrum disorder (ASD), motor development, sensory integration, sensory-motor rehabilitation, sensory motor room

## Abstract

*Background:* Motor skills in early and middle childhood are essential for physical play, social interactions, and academic development. Children with autism spectrum disorder (ASD) often exhibit atypical sensory responses, which can impact self-care and other developmental areas. This study explores the impact of sensory and motor rehabilitation using a Motor Sensory Room to stimulate motor development in children with ASD. *Methods:* Twenty-five children with ASD, preschool and school-aged (2–10 years), were divided into three groups based on the DSM-5 severity levels. The PEP-3 scale was used to assess cognitive, language, motor, emotional, social, and behavioral development. Ten children underwent a 3-month Motor Sensory Room intervention, and data were collected longitudinally. A control group of ten children, matched in age, sex, and diagnosis, did not receive the intervention. A 12-month follow-up is planned for all participants. *Results:* Children exhibited diverse profiles. Type B subjects displayed more severe symptoms, while Type A showed milder symptoms with better language and interpersonal skills. After the 3-month intervention, improvements were noted in several PEP-3 areas. For sensory levels (hypo-reactivity), the percentage of individuals at medium levels increased from 44% to 50%. For hyper-reactivity, the percentage at medium levels rose from 30% to 40%. Motor skills improved, with the percentage of individuals with high motor abilities rising from 20% to 25%. Relational behaviors also saw gains, with an increase from 50% to 55% in medium-level behaviors. The experimental group demonstrated better outcomes compared to the control group, particularly in sensory and motor skills. *Conclusions:* Preliminary findings suggest that sensory and motor training in a Motor Sensory Room improves sensory integration, motor coordination, and social interaction in children with ASD. Further research is needed to confirm long-term benefits.

## 1. Introduction

Symptoms related to sensory atypicality, as described by Miller [1], constitute a complex set of behavioral reactions to the sensory environment. These can be categorized into three primary models: hyperreactivity, hyporeactivity, and sensory-seeking. Hyperreactivity involves overreacting to the sensory environment (e.g., covering your ears to the sound of someone singing). Hyporesponsive behaviors are subreactions to the sensory environment (e.g., not addressing a loud sound). Examples of sensory-seeking behaviors include prolonged visual inspection of toys or repetitive touching of objects. Sensory responses are defined as the child’s reactions to sensory processing by the basic sensory systems (auditory, visual, vestibular, touch, smell, and taste) and are based on daily observations from a functional point of view. On the other hand, atypical sensory responses are unusual reactions to sensory stimuli, generally reported as hypo- and hyper-reactive reactions. Atypical sensory responses can interfere with self-care skills [2]. Along with the self-care skills already mentioned, there is an increasing number of children with ASD, cognitive, and sensory domains that interfere with their academic progress [3,4,5,6].

Recent estimates of the prevalence of sensory symptoms in people with autism spectrum disorder (ASD) range from 69% to 93% in children and adults and have been featured as a diagnostic criterion for ASD in the Diagnostic and Statistical Manual of Mental Disorders [7]. Despite the high prevalence of symptoms and their centrality in autism spectrum disorders (ASD), the developmental trajectory of sensory atypicalities is not entirely unknown. Some studies have explored the influence of a potential hereditary component, though they often used small samples and predominantly focused on mothers. The results highlight a very high percentage of mothers of individuals with autism exhibiting atypical sensory processing scores [8,9,10].

The relationship between sensory symptoms and chronological age is debated. Some studies on Sensory Over-Responsivity (SOR) have found that it often emerges in early childhood [11,12], followed by steeper increases that differentially predict more autistic traits at school age [13] and are associated with elevated anxiety as well as internalizing and externalizing problems [14], which may improve with age [15].

Unusual sensory behaviors can potentially interfere with adaptive functioning, but the relationship between these constructs is currently unclear in ASD. Qualitative interviews with parents reveal those sensory symptoms that limit participation in family routines and activities [16]. Higher rates of sensory behaviors are also linked to family and parent stress. The relationship between sensory symptoms and adaptive functioning may depend on the age of the participants, the domains of adaptive functioning and sensory processing compared, and the inclusion of cognitive abilities as an additional covariate [17,18].

The level of sensory dysfunction distinguishes preschoolers with ASD from those with subthreshold ASD and contributes to homogeneity within the autism spectrum. Understanding the risk factors and clinical correlates of sensory dysfunction in early childhood can strengthen intensified screening and precision therapies to support development and reduce worrisome symptoms later in life. However, little is known about risk factors for sensory dysfunction in young children [19].

During early and late childhood, children also use their fine motor skills to explore the environment, engage in physical play, initiate social interactions, and develop basic academic skills. Studies on general and fine motor skills in children with ASD are scarce but have shown motor delays, difficulties with motor coordination, imitation of body movements, motor planning, perceptual–motor integration, and postural control [20]. It is important to add and highlight the presence of motor deficits in children with ASD reaching 50–85% [7]. In addition, the lack of studies on motor skills impairments in children with ASD is not so clear as it may seem, as some studies have found children with ASD show a variety of motor impairments other than those already mentioned in this study, including poor bilateral coordination, reduced grip strength, impaired motor speed, gait abnormalities, and poor fine-motor control (e.g., manual dexterity, handwriting, object control) [21].

Current DSM-5 [7] and ICD-11 [22] guidelines allow clinicians to assign a concurrent diagnosis of developmental (motor) coordination disorder (DCD) for autistic individuals with significant motor problems. Poor motor skills with the onset of symptoms in the early stages of development characterize DCD. Studies have shown considerable overlap in the motor behavioral characteristics seen in autism and DCD. However, others indicate that motor problems in autism and DCD may result from several underlying sensorimotor mechanisms [23].

Sensory rooms are structured environments designed to stimulate the senses through lights, sounds, textures, and movement. For children with ASD, these rooms offer an opportunity to improve motor skills, sensory regulation, and social interaction and are effective in improving sensory responses like taste, smell, tactile behaviors, and motor skills [24,25]. Through controlled and personalized stimuli, sensory rooms help reduce hyper- or hypo-sensory reactivity, promoting greater stability and responsiveness to stimuli. Intervention in these rooms can also improve motor and relational skills, supporting the child’s overall development.

Sensory rooms are commonly used as part of therapeutic programs for children with autism, based on studies demonstrating improvements in motor and sensory abilities. A notable example is the study by Alvarez-Fernandez and colleagues [26] confirmed that regular exposure to targeted sensory stimuli can increase tolerance to environmental factors and promote better motor balance. The studies of Essary and colleagues [27] and Park and colleagues [28], describe all the processes of creation, building, and evaluation of sensory adaptive environments in school settings. Likewise, using a single case design, Hill and colleagues [29] found the use of sensory adaptive environments reduced autistic symptoms and increased the engagement of 14-year-old girls with a diagnosis of autism living in a residential school, demonstrating promise for the use of these resources in institutional settings to support residents.

A recent study referred to the incorporated technology to detect and respond to needs in real-time [30].

Given these theoretical-clinical premises, the present preliminary study, through the presentation of the educational-rehabilitative training activities within the sensory-motor room, compares the pre- and post-training data carried out with 10 children with autism in early and middle childhood to assess the effectiveness of the procedure used already in progress. In addition, the data of the experimental group were compared with the control group.

## 2. Materials and Methods

### 2.1. Procedure

The procedure used is divided into several specific phases of assessment and training. In the assessment phase, a diagnostic evaluation for ASD is conducted at accredited public facilities (ASL SALERNO) through the administration of the Autism Diagnostic Observation Schedule (ADOS-2) [31] and Autism Diagnostic Interview-Revised (ADI-R) [32] by specialized personnel (child neuropsychiatrists and psychologists).

The ADOS is a structured assessment that involves direct observation of the person in social interaction and communication situations. It is performed in different contexts and is used to identify behaviors and signs associated with autism. It consists of a series of modules, chosen according to the person’s age and language level, and assesses communication, social interaction, use of play, and repetitive or restricted behavior. We have used modules 1-2-3.

The ADI-R is a structured clinical interview designed to assess symptoms of ASD through the involvement of parents or caregivers. It consists of 93 questions that focus on three main areas: (1) quality of social interaction (e.g., emotional sharing, response to others), (2) communication and language (e.g., social use of language, pronoun reversal), and (3) repetitive behaviors and interests (e.g., unusual preoccupations, sensory interests). It is used to identify behaviors associated with ASD and to better understand the individual’s clinical profile.

Following the initial assessment, children are grouped based on their clinical severity, reflecting the intensity of support required. This approach aligns with the DSM-5’s dimensional framework, emphasizing individual variability rather than rigid categorical distinctions. Specifically, the classification considers two core dimensions: (1) the degree of impairment in social communication and interaction and (2) the presence and severity of repetitive and restricted behaviors. Grouping by severity not only enhances the ability to tailor interventions to the unique needs of each child but also facilitates the identification of trends associated with different levels of impairment. Additionally, this methodology ensures adherence to established diagnostic frameworks, promoting consistency and comparability across analyses.

Specifically:

- Level 1: “requires some support” (Group A),
- Level 2: “requires substantial support” (Group B).
- Level 3: “requires very significant support” (Group C).

We will have 3 different types of clinical-diagnostic profiles in various areas of functioning (language and communication, sensory reactivity, sphincter control, and self-care, relational reactions). In particular:

- Type A, which presents milder difficulties,
- Type B, which shows greater severity in attitudinal and relational domains, and
- Type A/B, which falls in an intermediate position.

### 2.2. Participants

Twenty-five children (24 boys and 1 girl) participated in this study (experimental group), age 2–10 years.

Based on the assessment and diagnostic criteria described above, the experimental group is formed:

- 40% from Type A
- 30% from Type B
- 30% from Type A/B

All children practice ABA rehabilitation interventions, psychomotor skills, and speech therapy.

For our study and to better monitor the effectiveness of the training conducted in the sensory and motor room, 10 children from the sample formed the Experimental Group, undergoing the 3-month training, and their performances were compared with the Control Group, made up of 10 children (similar in age, gender, and diagnosis) who did not undergo the specific training.

### 2.3. Motor Sensory Room Training

The design of a sensory room focuses on creating a multi-sensory environment (MSE) that combines visual, auditory, tactile, and sometimes olfactory elements to provide a calming and engaging space for individuals with sensory needs. A typical sensory room might include adjustable lighting systems, such as fiber optics or bubble tubes; soft furnishings to promote relaxation; textured surfaces to stimulate touch; and sound equipment to play soothing or adjustable sounds. In Snoezelen^®^ rooms, sensory input is often tailored to the individual, enabling them to interact and control their experience, which is particularly beneficial for children with autism spectrum disorder (ASD) [24].

Examples of sensory rooms include the Snoezelen^®^ rooms developed in the Netherlands, which have become globally recognized for their therapeutic applications. Additionally, sensory rooms inspired by the Autism ASPECTSS™ model are designed with features like acoustic control, sensory zoning, and escape spaces to accommodate the unique needs of autistic individuals [33]. The hybrid approach described by Robles-Bykbaev and colleagues [34] integrates sensory rooms with robotic assistants and ontologies to enhance therapeutic interventions for children with ASD, showcasing innovative uses of these environments.

Sensory rooms are widely implemented in countries that emphasize ASD-inclusive education and therapy, such as the Netherlands, the United Kingdom, and the United States. These environments are increasingly recognized as effective tools for managing sensory challenges and promoting well-being in individuals with ASD [35].

In our study, the Sensory Motor Room is conceived as a welcoming and safe space, designed to offer children an environment for exploration and learning, where they can develop their skills in an inclusive and fun context. Through a variety of interactive elements and engaging activities, this room offers a multi-sensory experience that promotes children’s sensory development and motor skills. All the activities are described in Table 1.

**Figure 1:**
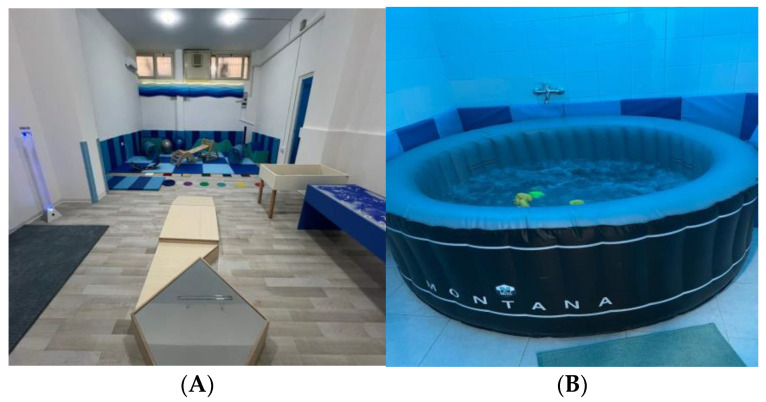
The motor sensory room. (**A**) Motor Sensory Room for sensory and motor skill development in children with ASD. (**B**) Hydrotherapy pool for sensory stimulation and relaxation in children with ASD.

Figure 1 of the motor sensory room highlight its interactive and modular features, while the positive effects include improvements in attention, reductions in repetitive behaviors, and enhanced motor skills (see Table 2).

The effectiveness of the training is evaluated using the standardized test Psychoeducational Profile (PEP-3) [36], a widely used assessment tool for assessing the development of preschool and school children with learning or developmental difficulties. It provides a clinical measure of the child’s abilities across key areas through a combination of subtests, direct observation, and parent questionnaires. The PEP-3 assesses verbal/pre-verbal cognition, expressive and receptive language, fine and gross motor skills, and visuo-motor imitation, as well as emotional expression, social reciprocity, and characteristic behaviors. Additionally, integrating the sensory evaluation section of the PEP-3 with data from other standardized tools such as ADOS-2 [31] and ADI-R [32] enables the identification of specific patterns of hyperreactivity, hyporeactivity, or sensory-seeking behaviors. In our case, these insights were further refined by cross-referencing the sensory findings with observed behaviors assessed through the PEP-3, providing a comprehensive understanding of the child’s sensory and behavioral profile to inform targeted educational and therapeutic interventions [1,2,3,4,5,6].

To determine whether a subject exhibits hyposensitivity (HYPO) or hypersensitivity (HYPER), specific sections of the PEP-3 were used to assess responses to visual, auditory, tactile, gustatory, and olfactory stimuli. During the evaluation, the subject is exposed to sensory stimuli (e.g., bright lights, sounds, textured surfaces, strong odors, etc.). The response level to each stimulus is recorded based on categories such as Low, Medium, and High, which correspond to the following:

- Hyposensitivity (HYPO): reduced or absent reactions to stimuli.
- Typical Response (Normotypical): appropriate and proportional reactions.
- Hypersensitivity (HYPER): heightened or disproportionate reactions to stimuli.

If a sensory category shows a predominant occurrence of hypo- or hyper-sensory responses, that sensory domain can be considered critical. The values reflecting the overall distribution of responses in the experimental sample across all sensory categories of the PEP-3 indicate 25.82% hyposensitivity and 32.42% hypersensitivity.

### 2.4. Data Analysis

All data were analyzed with IBM SPSS v. 29 software.

The data were analyzed using cross-tabulations to examine the descriptive profiles of the three diagnosis types (A, B, and A/B) and the percentage differences in the pre-post test.

We also performed a descriptive comparison between the experimental group and 10 other children, homogeneous in sex, age, and diagnosis to the experimental group, who did not train in the sensory room.

## 3. Results

The compared data at the pre-test vs. post-test of the 10 children, all with diagnosis A/B level, who underwent the 3-month training in the motor sensory room, show different aspects.

**Scheme 1:**
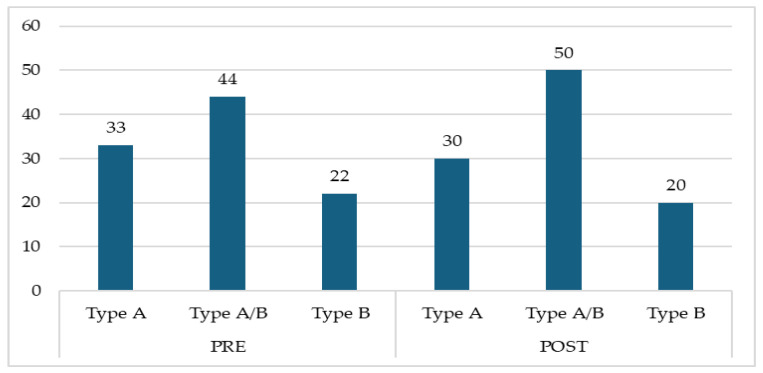
Pre- and post-test (%) for the Sensory Level (HYPO-SENSITIVITY).

**Scheme 2:**
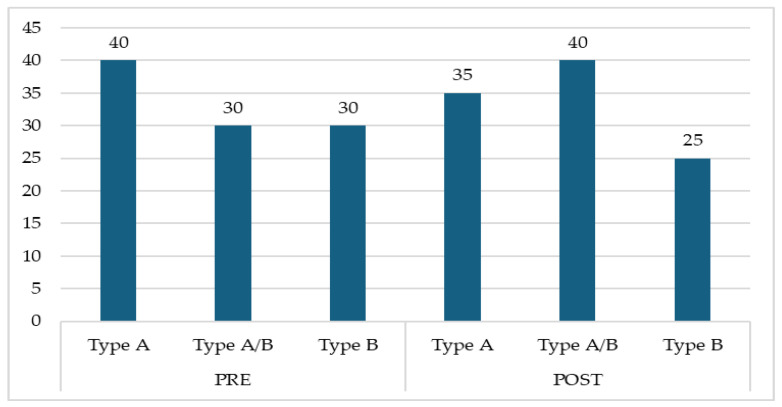
Pre- and post-test (%) for the Sensory Level (HYPER-SENSITIVITY).

Scheme 1 and Scheme 2 show the HYPO and HYPER sensory levels, in percentage, across the three different groups of children, identified as “Type A”, “Type B”, and “Type A/B”, both before (PRE) and after (POST) the training in the Motor Sensory Room. Specifically, 50% of participants reported Type A/B sensitivity in the HYPO condition and 40% in the HYPER condition. For Type A, 30% of participants reported this sensitivity in the HYPO condition, compared to 35% in the HYPER condition.

Type B sensitivity was reported by 20% in the HYPO condition and 25% in the HYPER condition. The pre-test and post-test data for the Type A/B children highlight various aspects: in the HYPO Sensory Level category (see Scheme 1), although the percentage of individuals with high sensory levels decreased from 33% to 30%, there was a significant increase in those at medium levels, rising from 44% to 50%, suggesting an increase in sensory stability. In the HYPER Sensory Level category (see Scheme 2), the percentage of individuals with high levels declined from 40% to 35%, while medium levels increased from 30% to 40%, indicating improved tolerance to hyper-reactive stimuli.

**Scheme 3:**
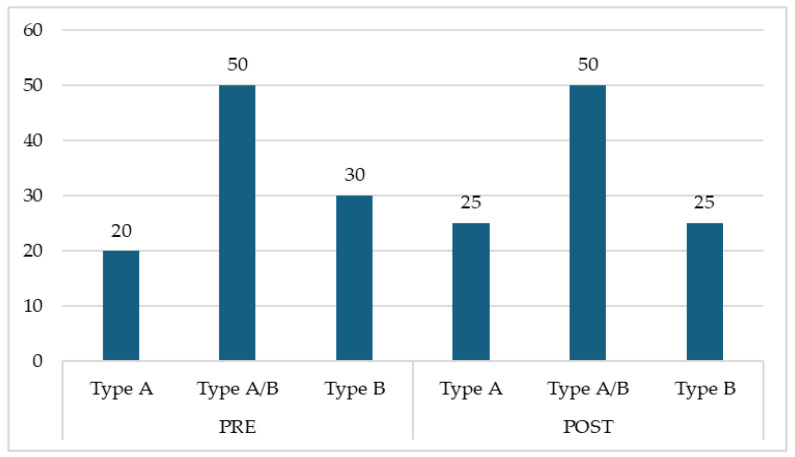
Pre- and post-test (%) for the Compromised Motor Skills.

Scheme 3 represents motor skill impairments (in percentage) across the three groups of children, defined as “Type A”, “Type B”, and “Type A/B”, both PRE- and POST-training. It is noticeable that there was an increase in the percentage of individuals with high motor skills, rising from 20% to 25%, while those with medium motor skills remained at 50%. The percentage of individuals with low motor skills decreased from 30% to 25%, reflecting progress in motor abilities.

**Scheme 4:**
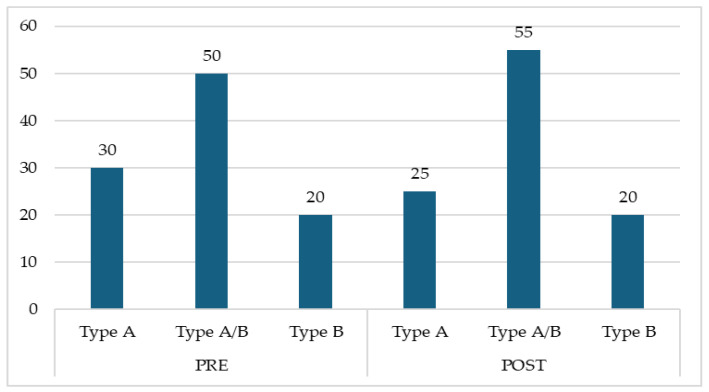
Pre- and post-test (%) for the Limited Relational Behaviors.

Finally, regarding Limited Relational Behaviors (see Scheme 4), the percentage of individuals with high levels of limited relational behaviors decreased from 30% to 25%, while the percentage of those with medium levels of limited relational behaviors increased from 50% to 55%.

**Scheme 5:**
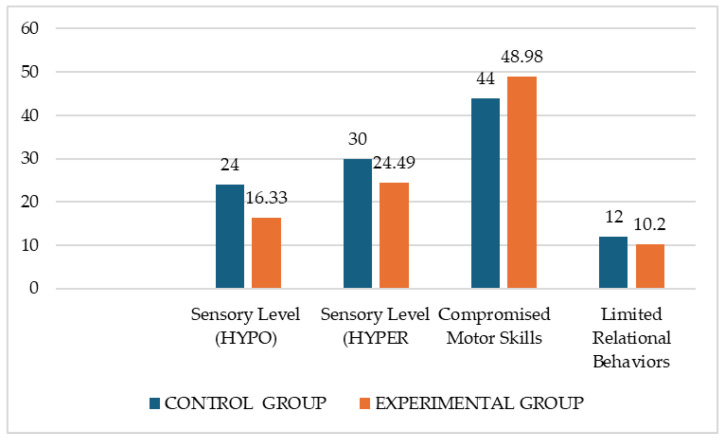
Comparison of Experimental Group with Control Group.

We also compared the children who completed the 3 months of experimentation in the motor sensory room and the group that did not complete the training, i.e., the control group (see Scheme 5).

As we can see, Scheme 5 compares sensory and motor responses between children who have not participated in sensory-motor training (Control Group) and those who have (Experimental Group).

The comparison of the Experimental group and Control Group data also highlights improvements. The Control Group shows higher hypo-sensitivity at 24% compared to 16.33% in the Experimental Group, indicating a potential lack of sensory awareness. The Control Group also exhibits higher hyper-sensitivity at 30% versus 24.49% of the Experimental Group. The Experimental Group has lower compromised motor skills at 44% compared to 48.98%, suggesting the beneficial effects of training. Both groups show similar levels of limited relational behaviors, with 12% in the Control Group and 10.20% in the Experimental Group, emphasizing the need for training for further social skills development.

## 4. Discussion

The findings from this study provide important insights into the profiles of individuals with different diagnostic types (A, B, and A/B) across various domains of functioning, including language, sensory processing, and behavior. These results align with previous research in neurodevelopmental disorders, highlighting the heterogeneity of symptomatology within the spectrum [7,37,38].

The observed differences in language abilities across the diagnostic types, according to the assessment tools applied, suggest that individuals with Type B diagnoses often present with more pronounced language impairments than those with Type A profiles. Communication difficulties range from mild to severe, with some children being verbal, while others remain non-verbal or have very limited language abilities. This diversity in profiles stems from the presence of comorbid factors, the intrinsic nature of ASD, or a combination of both, often accompanied by complex clinical symptoms [39]. This finding underscores the need for tailored language interventions targeting the specific needs of each diagnostic group [40].

The patterns of sensory processing observed in the current study align with the growing research on atypical sensory processing in neurodevelopmental disorders [41,42,43,44,45,46,47].

The diversified hyper- or hypo-reactive profiles across the diagnostic types highlight the importance of comprehensive sensory assessments and the development of individualized sensory-based interventions [48].

The differences in behavioral and relational reactions among the diagnostic types are consistent with the literature on the social-emotional challenges associated with neurodevelopmental disorders [49]. In the future, we will explore how those social-emotional challenges may negatively affect the quality of life and well-being of those who suffer from neurodevelopmental disorders and highlight some promising practices to mitigate them [50,51,52]. The more severe attitudinal and relational difficulties observed in Type B individuals underscore the need for targeted social skills training and emotion regulation interventions.

The findings from this study not only contribute to our understanding of neurodevelopmental disorders but also have significant implications for the mental health of children with autism and their families.

Children with autism often struggle with sensory hypersensitivity, motor skills deficits, and relational difficulties, which can significantly impact their mental health. Research has shown comorbidity with anxiety, stress, and behavioral issues in children with ASD. Additionally, children with autism often face language impairments, especially those with more severe diagnostic types, such as Type B, as noted in this study. The development of expressive language during the first year of life is slower compared to language comprehension, and it is also known that gestures, non-verbal cognitive ability, and response to joint attention are significant predictors of receptive and expressive language [53]. Language barriers can hinder social interactions, leading to frustration, isolation, and emotional distress. The differences in social-emotional challenges, including difficulties with emotional regulation and social interactions, also contribute to higher levels of anxiety and depression in children with ASD.

The challenges faced by children with autism inevitably extend to their families. Parents of children with autism experience higher levels of stress, anxiety, and depression compared to parents of neurotypical children. The unpredictable nature of sensory sensitivities, the need for constant behavioral management, and difficulties with communication can lead to feelings of frustration and helplessness. Studies have shown that families often experience emotional and physical exhaustion, as well as financial strain, due to the need for specialized therapies and interventions [54].

In particular, the relational difficulties observed in type B individuals, which manifest as more severe social and emotional challenges, can strain family relationships. Siblings and parents may struggle to engage with the child, leading to feelings of isolation within the family unit. This can create a vicious cycle of stress that exacerbates the mental health issues of both the child and the caregivers [55].

Given the complex needs of children with ASD, continuous intervention and support over longer periods (such as the planned longitudinal analysis) will be crucial in providing a more complete picture of the long-term impact of therapeutic strategies.

The motor sensory room training has improved relational reactions, reduced provocative behavior, and enhanced sensory responsiveness. Nonetheless, persistent challenges, particularly in sensory hypersensitivity, motor skills, and personal autonomy, underscore the need for continued intervention and support. Tailored strategies focusing on these areas will ensure holistic development and well-being for these children. Even if only 3 months of training, the results are encouraging.

The observed lack of significance in the results may be influenced by specific characteristics of the sample, which could have introduced variability or limited the generalizability of findings. For instance, factors such as the small sample size, heterogeneity in age or developmental profiles, or variations in prior therapeutic exposure might have played a role. These elements can lead to insufficient statistical power, making it challenging to detect subtle but meaningful effects.

Additionally, the experimental context itself is still in a developmental phase, with methodologies and protocols being fine-tuned based on these preliminary outcomes. This adaptive approach, while valuable for long-term precision, may introduce inconsistencies in the short term, particularly when interventions are adjusted to meet individual needs.

Another potential limitation is the reliance on a single method of assessment for measuring outcomes, which might not fully capture the nuanced changes induced by the intervention. Broadening the scope of evaluation tools or incorporating multimodal data could improve the sensitivity of future studies. These limitations highlight the need for iterative refinement of the experimental framework, ensuring that subsequent research builds on these foundational findings to achieve more robust and generalizable results.

The planned longitudinal analysis of the Sensory Room, after one year of training, will provide valuable insights into the differential impact of this approach on the symptomatology of the various diagnostic groups. This information can guide the development of more effective and tailored therapeutic strategies [56,57].

## 5. Conclusions

The conclusions of this study offer a general perspective on the implications of the work carried out, identifying possible practical applications and potential future developments. Despite the limitations, the conclusions highlight the importance of the topic dealt with and pave the way for further investigations and insights, which we are going to carry out over 12 months of experimentation.

## Figures and Tables

**Table 1:** Activities of motor sensory room training.

Sensory Stimulation Activities	Use colored lights and projectors to create plays of light and shadow that can be followed with the eyes.
	Provide a variety of tactile surfaces with different textures (e.g., soft, rough, smooth) to encourage tactile exploration.
	Offer sound and musical toys that can be touched or operated to stimulate hearing and encourage active participation.
	Use light and pleasant aromas through diffusers or natural essences to stimulate the sense of smell in a non-invasive way.
	Incorporate elements of movement, such as swings, to stimulate the vestibular sense and improve the sense of balance.
Sensory Integration Activities	Use structured activities that engage multiple senses at once, such as combining sound and light with coordinated movements.
	Incorporate games that require the coordinated use of multiple senses, such as following auditory instructions to perform specific motor actions.
	Create sensory pathways that encourage your child to use all of their sensory abilities to overcome motor and cognitively stimulating challenges.
Motor Development Activities	Offer structured play activities encouraging body movement, such as jumping on soft surfaces or running through an obstacle course.
	Use motor equipment such as sensory mats, exercise balls, and unstable platforms to improve balance and coordination.
	Provide opportunities to imitate motor movements through role play and educator-led activities.
	Incorporate elements of cooperative play that require social interactions and motor collaboration between children.
Sensory Regulation Activities	Teach sensory self-regulation techniques, such as deep breathing or rhythmic movement, to help children manage their sensory reactions.
	Create a safe and quiet space within the motor sensory room where children can retreat if they feel overwhelmed by sensory stimuli.
	Use guided relaxation activities like guided meditation or visualization to help children find calm and sensory balance.

**Table 2:** Summary of the positive effects of motor sensory room training.

Improvements in Attention	Enhanced focus and sustained engagement during activities
Reductions in Repetitive Behaviors	Decrease in stereotyped motor movements and vocalizations
Enhanced Motor Skills	Development of gross and fine motor abilities through controlled sensory input
Reduced Anxiety and Agitation	Creation of a calming environment that minimizes sensory overload
Improved Self-Regulation	Increased ability to manage sensory responses independently

## Data Availability

The datasets used during the current study are available from the corresponding author upon reasonable request.
